# Investigating the association of mechanical restraint with somatic harmful outcomes: national register-based study

**DOI:** 10.1192/bjo.2024.799

**Published:** 2024-11-11

**Authors:** Lone Baandrup, Marie Kruse

**Affiliations:** Department Bispebjerg-Gentofte, Mental Health Centre Copenhagen, Denmark; Centre for Neuropsychiatric Schizophrenia Research, Mental Health Centre Glostrup, Denmark; and Department of Clinical Medicine, University of Copenhagen, Denmark; Danish Center for Health Economics, University of Southern Denmark, Denmark

**Keywords:** Coercion, mechanical restraint, adverse events, cohort study, harmful outcomes

## Abstract

**Background:**

Coercive measures to manage disruptive or violent behaviour are accepted as standard practice in mental healthcare, but systematic knowledge of potentially harmful outcomes is insufficient.

**Aims:**

To examine the association of mechanical restraint with several predefined somatic harmful outcomes.

**Method:**

We conducted a population-based, observational cohort study linking data from the Danish national registers from 2007 to 2019. The primary analyses investigated the association of mechanical restraint with somatic adverse events, using panel regression analyses (within-individual analysis) to account for repeated exposures and outcomes. Secondary between-group analyses were performed with a control group exposed to types of coercion other than mechanical restraint.

**Results:**

The study population comprised 13 022 individuals. We report a statistically significant association of mechanical restraint with thromboembolic events (relative risk 4.377, number needed to harm (NNH) 8231), pneumonia (relative risk 5.470, NNH 3945), injuries (relative risk 2.286, NNH 3240) and all-cause death (relative risk 5.540, NNH 4043) within 30 days after mechanical restraint. Estimates from the between-group analyses (comparing the exposed group with a control group of 22 643 individuals) were non-significant or indicated increased baseline risk in the control group. A positive dose–response analysis for cardiac arrest, injury and death supported a causative role of mechanical restraint in the reported associations.

**Conclusions:**

Although the observed absolute risk increases were small, the derived relative risks were non-negligible considering that less restrictive interventions are available. Clinicians and decision makers should be aware of the excess risk in future decisions on the use of mechanical restraint versus alternative interventions.

Aggressive or violent behaviour is a frequent clinical challenge in psychiatric treatment settings, with an estimated mean prevalence of 17% (ranging from 3 to 44%) in patients admitted to acute psychiatric wards in high-income countries.^1^ Coercive measures are considered necessary interventions in mental healthcare, to prevent patients from posing a danger to themselves or others when disruptive or violent behaviour cannot be managed by other less restrictive approaches. The use of coercion is regulated by national law, and therefore variation exists in the range of available coercive measures and in what situations they are permissible.^2,3^ During recent decades, an increasing focus on the prevention of the use of coercion has evolved with systematic interventions in psychiatric wards to provide a more secure environment, with a focus on reducing obvious trigger points arising from unnecessary habits of staff behaviour, a more patient-focused organisation of the wards, improved patient/staff ratio, response teams and changes in attitude and culture.^4–7^ Recent experimental evidence has pointed to the superior effectiveness of training staff in verbal and non-verbal de-escalation techniques to effectively reduce the incidence and severity of aggression and the use of restraint in psychiatric units.^8,9^ Despite this, coercion remains a common practice,^10^ and efforts to reduce specific kinds of coercion have sometimes led to an increase in others.^11,12^ Recently, the World Health Organization and the World Psychiatric Association have joined to provide common goals for action regarding implementation of alternatives to coercion in mental healthcare.^13^

## Overview of physical harm in relation to coercion

It has long been recognised that physical harm and even death have been observed in relation to the use of coercive measures. A recent review that collected information from published data found that published studies were mainly case reports or case series, and that death was the most frequently reported harm, followed by venous thromboembolism (VTE) and injuries.^14^ The most frequent causes of death in the published studies were cardiopulmonary arrest (with a possible, but not well-described link to asphyxia resulting from pressure on the thorax or the position) and asphyxia caused by strangulation. The second most frequently reported harm in the included studies was deep venous thrombosis (DVT), which occurred in up to 11.6% of mechanically restrained patients despite prophylaxis.^15^ Overall, the authors of the review found a significant lack of systematically collected data on the incidence of adverse events related to coercive interventions. Another review, focusing on both seclusion (where the patient is confined to a locked room) and restraint,^16^ concluded that there was consistent evidence for negative effects, including development of post-traumatic stress disorder and increased length of hospital stay after seclusion, and DVT with restraint. A third recent systematic review concluded that it was not possible to determine which of the two interventions, mechanical restraint or seclusion, was superior, but that both measures were associated with pros and cons.^17^ Specifically for mechanical restraint, a superior effect on objective outcome measures was reported regarding the duration of coercion and the need to change the coercive intervention. However, regarding subjective outcome measures, seclusion was preferred, being considered less intrusive and more acceptable than mechanical restraint.^17^ Mechanical restraint is considered the most intrusive coercive practice,^2^ and must be expected to be associated with the highest risk of somatic harm because of the practical procedures and the immobile status of the mechanically restrained patient.^14^

In summary, a more complete knowledge of the risk of potential physical harms associated with mechanical restraint is mandatory to ensure the least possible harm to patients who are admitted to hospital because of mental illness.

## Aim of the study

This study aimed to investigate the association of mechanical restraint with somatic harmful events in adult patients, using the nationwide Danish registers. Seclusion is not allowed in Denmark, and therefore not evaluated in this study.

## Method

### Definition of the study population, exposure and outcomes

In Denmark, all use of healthcare services is registered in the National Patient Register (NPR)^18^ and all hospital psychiatric contacts are registered in the Danish Psychiatric Central Research Register.^19^ There were time-series breaks in these registers in early 2019, therefore only admissions starting before the 1 January 2019 were included in this study. All-cause mortality was analysed until 31 December 2019, and somatic outcomes were analysed until 31 January 2019. Furthermore, a complete national register covering all coercive episodes according to the Danish Mental Health Act is available for research purposes, as the National Register of Coercive Measures in Psychiatric Units.^20^

Physical restraint exists in several forms, and here we differentiate between mechanical restraint when a leather belt is used to strap the patient to the bed, and manual restraint when staff intermittently immobilises a patient by holding them manually.^14^ The Danish Mental Health Act allows several types of coercive measures when no other treatment options are available. We included the following measures of coercion: compulsory admission, involuntary treatment, manual restraint, forced sedative medication for acute tranquillisation and mechanical restraint.

This is a retrospective cohort study of adult individuals admitted to a hospital psychiatric department once or more, in Denmark between 2007 and 2018, who had at least one psychiatric admission starting after 2006 and before 2019, and who were exposed to coercion of any kind. Patients admitted before 2019 but who were still in hospital after 1 January 2019 were followed until the end of January 2019. Analysis of mortality was conducted until the end of 2019.

Data from the above-mentioned registers were linked using the Danish personal identification number assigned to all inhabitants at birth or immigration. Data from the Danish national registers are complete for all Danish inhabitants regardless of where they have moved around the country.

Exposure was defined as each incident of mechanical restraint, and the period of increased risk of somatic harmful events was defined as 30 days after the incident of mechanical restraint. We defined the exposure period as 30 days to increase the probability that a somatic event was related to the incident of restraint. A shorter period (e.g. 2 weeks) would have introduced a risk of missing somatic events related to the incident of restraint but occurring or being documented later than 2 weeks. A longer period would have increased the probability that somatic events that were not related to the incident of mechanical restraint would have been attributed to the incident of restraint. The 30 days is a methodological choice based on the nature of the outcome measures selected for this study (see description below). Based on the proposed physiological mechanisms behind the harmful outcomes in focus, they are most likely to appear within the first couple of days to weeks after mechanical restraint. We prolonged the period to 30 days to ensure as complete as possible documentation of the events in the central registers. Despite this prolongation to 30 days, there will probably be a certain level of missed events owing to delay or lack of documentation. The exposure group was defined as individuals who had been mechanically restrained once or more in the study period 1 January 2007 to 1 January 2019. The control group was defined as individuals from the study population who had been exposed to any form of coercion other than mechanical restraint, including compulsory admission, involuntary treatment, manual restraint and forced sedative medication for acute tranquillisation. The exposed individuals could have been subject to these other methods of coercion as well. The definition of the control group was chosen to ensure comparable illness severity of the control group.

Data were analysed in encrypted form, and informed consent or ethical approval was not required according to Danish regulations.

### Statistical strategy

Upon division of the population into the exposed group (mechanical restraint) and controls, data were structured as an unbalanced panel, covering the period 1 January 2007 to 31 January 2019 (analysis of mortality until 31 December 2019). In this panel, a regression analysis was conducted. Please see the Supplementary Material available at https://doi.org/10.1192/bjo.2024.799 for detailed specifications of the statistical model. The choice of outcome measures was based on the previous literature.^14,16^

Since the regression coefficient is difficult to interpret in terms of clinical significance, we calculated the number needed to harm (NNH) and the relative risk in case of statistically significant findings in the regression analysis. The within-individual NNH denotes how many times an individual should be mechanically restrained to obtain one additional incident of somatic harmful outcome within 30 days after the incident of restraint, and the between-group NNH denotes how many individuals should be mechanically restrained to obtain one additional incident of somatic harmful outcome in the total follow-up period of the study. The within-individual relative risk estimates the relative risk of a somatic harmful outcome 30 days after being mechanically restrained compared with the days in the observation period not preceded by restraint within 30 days in the same individual. In other words, the observation period for each individual consists of one or more 30-day periods preceded by mechanical restraint and the remainder of the time classified as not exposed to harmful events after mechanical restraint. The within-individual relative risk thus estimates the risk of a harmful event after mechanical restraint compared with the risk of a harmful event in non-exposed periods in the same individual. The advantage of this panel model is that the same person is compared with themselves, thereby controlling for time-invariant variables that would otherwise confound the association under investigation. The between-group relative risk estimates the relative risk of a somatic harmful outcome in patients who were mechanically restrained compared with non-restrained patients in the observation period of the study.

For the main analysis, mechanical restraint was treated as a binary variable, because this was deemed the most feasible in a panel data analysis. However, we also wished to evaluate if longer durations of restraint were more harmful. We therefore conducted a dose–response analysis in the group of mechanically restrained patients, controlled for age, gender, substance misuse and psychiatric diagnoses. This analysis cannot be applied to the base-case analysis because the control group had a dose of zero.

## Results

The data-set comprised data from 35 665 individuals, 13 022 of whom were mechanically restrained. Individuals contributed data daily, resulting in 53 372 754 observations for the restrained group and 103 698 093 observations for the control group. Individuals were censored on 31 January 2019, or at the time of death if this occurred before the end of 2019.

Table 1 shows the demographics of the study population as characterised by entry into the cohort. Individuals in the exposed group were predominantly male (66%), and the mean age was 36.7 years in the exposed group and 46.1 years in the control group. The predominant diagnosis in both groups was schizophrenia and other psychotic disorders. In Table 1, both age at first coercion event and age at first psychiatric diagnosis are shown. The table covers the period 2007–2019, such that all events of death are included.

**Table 1:** Demographic and clinical characteristics of the study cohort

	Restrained group *n* = 13 022	Control group *n* = 22 643
Male, *n* (%)	8608 (66.1%)	11 322 (50.0%)
Age at first coercion, mean (s.d.), years	45.784 (16.666)	53.111 (19.394)
Number of incidents of mechanical restraint per individual, median (IQR)	1 (2)	−
Median duration of mechanical restraint in days (IQR)	2 (1)	−
Mean age at first psychiatric admission, mean (s.d.), years	36.728 (17.724)	46.065 (20.833)
Number of patients with a diagnosis F00–F09 Organic mental disorders, *n* (%)	3073 (23.6%)	6091 (26.9%)
Number of patients with a diagnosis F10–F19 Mental and behavioural disorders due to psychoactive substance use, *n* (%)	7084 (54.4%)	8333 (36.8%)
Number of patients with a diagnosis F20–F29 Schizophrenia, schizotypal and delusional disorders, *n* (%)	7826 (60.1%)	11 299 (49.7%)
Number of patients with a diagnosis F30–F39 Mood disorders, *n* (%)	5261 (40.4%)	8808 (38.9%)
Number of patients with a diagnosis F40–F49 Neurotic, stress-related and somatoform disorders, *n* (%)	5899 (45.3%)	9465 (41.8%)
Number of patients with a diagnosis F50–F59 Behavioural syndromes associated with physiological disturbances and physical factors, *n* (%)	443 (3.4%)	679 (3.0%)
Number of patients with a diagnosis F60–F69 Disorders of adult personality and behaviour, *n* (%)	3464 (26.6%)	4212 (18.6%)
Number of patients with a diagnosis F70–F79 Mental retardation, *n* (%)	755 (5.8%)	838 (3.7%)
Number of patients with a diagnosis F80–F89 Disorders of psychological development, *n* (%)	469 (3.6%)	543 (2.4%)
Number of patients with a diagnosis F90–F99 Behavioural and emotional disorders with onset usually occurring in childhood and adolescence, *n* (%)	4050 (31.1%)	4710 (20.8%)
Number of patients who died 2007–2019, *n* (%)	4597 (35.3%)	9352 (41.3.3%)

Patients could have more than one diagnosis. Diagnosis codes refer to ICD-10. IQR, interquartile range.

Supplementary Table 1 lists the distribution of coercive interventions in the study population. The exposed group was subjected to involuntary treatment and manual restraint more than the control group, but this was less so for compulsory admission. It is worth mentioning that although many individuals were subjected to restraint more than once, the median number of restraints per individual in the exposure group across the observation period was 1. The results of the panel regression (within-individual analyses) are shown in Table 2, together with the secondary estimates of the between-group analyses. Both crude and adjusted results are presented for the between-group analyses, whereas the within-individual analyses are already adjusted for time-invariant covariates and so only crude results are presented. Results of the full model panel regression can be found in Supplementary Table 2.

**Table 2:** Panel regression analysis of the association of mechanical restraint with somatic outcomes (for the full model and results see Supplementary Table 2)

		Coefficient	*P-*value	NNH	Relative risk
Thromboembolism					
Constant	Crude	0.0000477	0.005	−	−
Mechanical restraint – within-individual	Crude	0.000161	**0.040**	8231	4.377
Mechanical restraint – between-group	Crude	−0.0000127	0.592	−	−
Mechanical restraint – between-group	Adjusted	−9.80 × 10^−7^	0.969		
Pneumonia					
Constant	Crude	0.0000928	<0.0001	−	−
Mechanical restraint – within-individual	Crude	0.0003111	**<0**.**0001**	3945	5.470
Mechanical restraint – between-group	Crude	−0.0000142	0.670	−	−
Mechanical restraint – between-group	Adjusted	−0.0000199	0.611		
Cardiac arrest				−	−
Constant	Crude	6.20 × 10^−6^	0.143	−	−
Mechanical restraint – within-individual	Crude	0.0000766	0.116	−	−
Mechanical restraint – between-group	Crude	4.74 × 10^−6^	0.276	−	−
Mechanical restraint – between-group	Adjusted	−0.0000102	0.162		
Injury				−	−
Constant	Crude	0.0002784	<0.0001	−	−
Mechanical restraint – within-individual	Crude	0.0004606	**<0**.**0001**	3240	2.286
Mechanical restraint – between-group	Crude	−0.0000822	0.115	−	−
Mechanical restraint – between-group	Adjusted	−0.000165	**0**.**036**	−	−
All-cause death				−	−
Constant	Crude	0.0003509	<0.0001	−	−
Mechanical restraint – within-individual	Crude	0.0002627	**<0**.**0001**	4043	5.540
Mechanical restraint – between-group	Crude	0.0000479	0.753	−	−
Mechanical restraint – between-group	Adjusted	−7.96 × 10^−7^	0.953	−	−

Statistically significant results are marked in bold. Adjusted results are adjusted for age at event, gender, psychiatric diagnoses, chronic somatic illness, substance misuse, year of event and municipality of residence. NNH, number needed to harm.

The risk of thromboembolism with mechanical restraint was statistically significantly increased in within-individual analysis (*β* = 0.000161, *P* = 0.04). The results indicated one additional episode of thromboembolism for every 8231 instances of mechanical restraint (within-individual NNH) and a relative risk of 4.377 (within-individual relative risk).

There was a significant association between mechanical restraint and pneumonia (*β* = 0.0003111, *P* < 0.0001). The results indicated one additional episode of pneumonia for every 3945 instances of mechanical restraint (within-individual NNH) and a relative risk of 5.470 (within-individual relative risk). For cardiac arrest, the corresponding result was not statistically significant (*β* = 0.0000766, *P* = 0.116). There was a statistically significant association of mechanical restraint with injury (*β* = 0.0004606, *P* < 0.0001). The results can be expressed as a within-individual NNH of 3240 and a within-individual relative risk of 2.286.

There was a statistically significant association of mechanical restraint with all-cause death (*β* = 0.0002627, *P* < 0.0001). The results can be expressed as a within-individual NNH of 4043 and a within-individual relative risk of 5.540.

The between-group estimates, representing the comparison between the mechanically restrained and the non-restrained group during the entire follow-up period, but independent of the timing of mechanical restraint, were statistically non-significant for all outcomes, except for injury (Table 2). For injury, the adjusted between-group analysis indicated (by a negative coefficient) that the control group had a higher baseline risk than the mechanical restraint group.

We conducted a dose–response analysis in the group of patients exposed to mechanical restraint, thus excluding the control group. The coefficients in Table 3 depict the risk increase per day of restraint. Here, a dose–response relationship was observed, i.e. increased duration of mechanical restraint significantly increased the risk of developing cardiac arrest (*P* < 0.0001), injuries (*P* < 0.0001) and death (*P* < 0.0001) within the 30-day interval after the incident of mechanical restraint (Table 3).

**Table 3:** Dose–response analysis of the association of duration of mechanical restraint with somatic outcome, for the restrained group

	Coefficient	*P-*value
Thromboembolism	2.27 × 10^−7^	0.173
Pneumonia	3.09 × 10^−7^	0.290
Cardiac arrest	2.20 × 10^−7^	**0**.**000**
Injury	3.26 × 10^−6^	**0**.**000**
Death	9.32 × 10^−7^	**0**.**000**

The coefficient indicates the increased risk of each somatic event per day the patient is restrained. Statistically significant changes are marked with bold.

## Discussion

In this register-based observational study based on a complete national data-set from 2007 to 2018, we report that the use of mechanical restraint was associated with an increased risk of somatic harmful outcomes, including thromboembolism, pneumonia, mechanical injury and all-cause death within 30 days of the coercive incident. These harmful outcomes are theoretically attributable to mechanical restraint via immobilisation, restricted ventilation when supine and physical interaction with the staff during the act of being forcefully restrained. In Denmark, all mechanically restrained patients are continuously observed by a staff member, and therefore accidental deaths caused by strangulation when slipping out of the belt (as reported in a German sample^21^) are prevented.

The results of this study represent an important contribution to the evidence base of the clinical consequences of the use of mechanical restraint. Up to now, most data on the topic have been drawn from case reports or small observational samples with a high risk of selection bias. Also hampering the validity of previous studies has been the lack of comprehensive consecutive data-sets covering all episodes of coercion, which is not available from many countries other than Denmark. A previous study found that across European countries, only Norway, Finland, Sweden and Denmark have comparable representative data on coercion.^2^

Our results confirm that mechanical restraint is associated with somatic harmful outcomes, which has previously been addressed in case studies and smaller observational designs based on consecutive series of mechanically restrained patients. When comparing the frequencies of somatic harmful outcomes in relation to mechanical restraint, VTE and death were the most consistently reported outcomes. Some studies reported injuries, but these studies focused on physical restraint and are therefore not directly comparable to the results on mechanical restraint from our study.^22,23^ The frequency of VTE in association with mechanical restraint has been reported with wide variation across published studies. In a retrospective chart review of 138 patients who were secluded and mechanically restrained,^24^ no cases of DVT were reported. In a review of 41 cases of VTE among 12 320 patients admitted to hospital, no association with mechanical restraint was reported.^25^ Takeshima et al^26^ investigated 1681 consecutive psychiatric in-patients and found the incidence of VTE in non-catatonic restrained patients to be 4.1%. In an observational study by Ishida et al of 181 mechanically restrained patients who were screened for DVT by measuring D-dimer in all patients when restraint was removed, a DVT (diagnosed by ultrasound in patients with increased D-dimer) incidence of 11.6% was observed despite pharmacological prophylaxis in two-thirds of the patients.^15^ The high incidence of DVT with mechanical restraint in the study by Ishida et al compared with other studies, including ours, is most probably caused by differences in diagnostic procedures and study design. In our data-set, VTE was diagnosed following usual clinical practice and not by use of screening procedures of all restrained patients. In the study by Ishida et al,^15^ all mechanically restrained patients were screened by measuring D-dimer, which means that more patients were detected than with standard clinical procedures. In addition, because of the lack of control group or control condition, which was a limitation of the design of the study by Ishida et al, the findings could not be compared with the prevalence of ultrasound-verified asymptomatic DVT in comparable patients without mechanical restraint. Thus, it is not possible to directly attribute the entire incidence of DVT in the study by Ishida et al to mechanical restraint, because the background incidence in the relevant population is unknown. Noteworthy, other studies have reported high incidence of VTE in psychiatric in-patients independent of restraint procedures.^25^ In a retrospective database review, Gaertner et al found a relatively high background incidence of VTE in psychiatric in-patients (3.32 per 1000 patients), being around 20 times higher than in the general population of the same age (mean age above 65 years).^25^ A high background incidence of VTE in psychiatric in-patients has been confirmed by Takeshima et al,^26^ who investigated 1681 consecutive psychiatric in-patients and found the incidence of VTE in non-catatonic unrestrained patients to be 1.2%.

Regarding mortality, another outcome reported across previous studies, the challenges with the smaller observational studies without a control group and with high variability in results become even more apparent. Pinninti and Rissmiller^27^ reviewed 1403 incidents of mechanical restraint and found no occurrences of death in relation to the restraint. Grover et al^28^ investigated cases with delirium, including 49 cases with mechanical restraint, and found that restraint before development of delirium and age <65 years were significant risk factors for increased mortality. Honkonen et al^29^ investigated the mortality of 3835 in-patients and found that mortality was increased (odds ratio 1.77) with use of coercive measures (i.e. mechanical restraint and other coercive practices) during the most recent hospital admission. However, the authors did not attribute the increased mortality to the use of coercive measures *per se*, but rather to a confounding by indication mechanism. Thus, our study supports previous findings of increased risk of death with mechanical restraint, and adds to the evidence base by providing a size of the association, as we included a control condition which other studies lack. In an observational study, there is always a risk of confounding by indication,^30^ i.e. that an observed association is not a result of the exposure but of an underlying condition that gives rise to the exposure. In the current study, this means that the increased risk of somatic harmful outcomes may not be a result of mechanical restraint *per se*, but of the characteristics of the patients subjected to mechanical restraint (e.g. poor health, poor dietary habits, smoking, etc.). As part of the study design (within-individual analyses), we controlled for such time-invariant confounders, which consequently did not affect the results. To emphasise this point, the dose–response analysis documented an increased risk of cardiac arrest, injuries and death with increased length of mechanical restraint. This finding supports the notion that the associations reported in this study might point to a causal mechanism of mechanical restraint and not merely confounding as a likely explanation of the findings. It is worth noting that the adjusted analysis did not support evidence of between-group differences – except for injury, where the control group was at higher risk. This apparent lack of increased risk with mechanical restraint in the between-group analysis may be interpreted as attributable to increased background risk of harmful outcomes in the study population. As such, the increased risk with mechanical restraint as observed in the within-individual analysis is likely disguised, and therefore not detectable in the between-group analysis across the study period (2007–2019).

### Strengths

Coercive interventions are meticulously controlled in Denmark and are subject to high levels of regulation. It is obligatory to register all coercive interventions in Denmark, and therefore this study is without selection bias regarding inclusion into the cohort. In clinical practice, the choice is not between mechanical restraint and no intervention, but between mechanical restraint and other less restrictive interventions. These less restrictive interventions will often include manual restraint and forced medication, but – dependent on the specific clinical situation – will typically also include de-escalating techniques and non-confrontative communication and staff behaviour, i.e. interventions that are not documented in the national registers. Consequently, the panel regression analysis ensures that the risk of somatic harmful events associated with mechanical restraint as documented in this study is not in comparison with any specific coercive measure that is limited to Danish law and clinical practice. Rather, the results indicate the excess somatic risk associated with mechanical restraint versus a set of unspecified interventions without mechanical restraint.

It is a strength of the study that we used panel regression analyses, because of the ability of these analyses to handle repeated exposures and outcomes in the same cohort. In a panel data-set, any unobserved characteristics of the individual that may affect findings are accounted for.

### Limitations

Somatic events during a psychiatric hospital stay are sometimes registered in the NPR at discharge, and data are therefore not always registered in real time. Therefore, survival analysis with time-to-event was not possible to conduct. This is an observational study and, as such, it is not possible to make a conclusion about causal relations. However, the results show a strong signal that mechanical restraint is associated with excess risk of somatic adverse events in patients drawn from a well-defined study population. It was not possible to compare with seclusion because of the lack of use of this intervention in Denmark. We were only able to include somatic harmful events of a certain severity that led to the registration of a separate diagnosis in the NPR. This will always happen when the patient is transferred for treatment in a somatic hospital. However, there is an actual risk that somatic harmful events treated as part of the psychiatric hospital stay were not registered in the NPR or were documented with a delay beyond 30 days (and thus not captured for this study) if the psychiatric hospital stay lasted longer than 30 days. This means that the results of this study, except for death (where data is always entered in real time), might be underestimations regarding the less severe cases of somatic harmful events, since the severe ones (including transference to a somatic hospital) will always be documented in real time. This imprecision in data entry is probably affecting the exposure periods more than the non-exposure periods, and might lead to a bias toward underestimation of events in the exposure periods. Thus, the frequency estimates presented in this paper must be regarded as minimum values. We were not able to include the medication received before or after the incident of mechanical restraint as a covariate, because medication administered during a hospital stay is not documented in the national registers. However, medication is not known to substantially increase the risk of the somatic outcomes in question, except for the risk of thromboembolism, and thus this limitation probably had little impact on the results.

Because of the register-based nature of this study, we did not include data on the possible harmful psychological consequences of mechanical restraint. The psychological impact of seclusion versus mechanical restraint has been evaluated in a small randomised controlled trial, which found no difference between these two coercive measures when using a subjective experience of coercive measures as outcome.^31^ However, at 1-year follow-up, the seclusion group reported fewer negative consequences, indicating that seclusion might be a less restrictive alternative for most patients.^32^

Mechanical restraint is used in clinical situations where a patient poses a danger to themselves or others. According to the Danish Mental Health Act, other less restrictive approaches (de-escalation, manual restraint and/or rapid tranquillisation) must have been applied and found insufficient, or the danger must be of such imminent nature that other less restrictive interventions are considered inappropriate to maintain safety for the patient in question and for co-patients and staff. A certain risk of somatic harmful events in relation to mechanical restraint is therefore generally accepted, and the question is whether the magnitude of the observed association of mechanical restraint with somatic harm is proportionate or disproportionate. Although we could be reassured that the absolute risk increase with mechanical restraint for each individual is low, it is difficult to argue that the excess risk is proportionate as long as less restrictive interventions are not fully implemented in clinical practice. A recent review of the alternatives to mechanical restraint in the management of agitation in psychiatric patients concluded that it is possible to reduce the use of restraints and coercive measures without increasing the number of incidents and violent behaviours.^33^ This can be done by applying non-invasive and non-pharmacological approaches, but more research is needed to compare available alternatives and to provide higher-quality evidence.^33^ Among other interventions, peer support in acute psychiatry may have the potential to prevent or reduce the use of restraint.^34^ In the current study, we examined the potential hazards for patients being exposed to mechanical restraint, but it is well known that the act of forcefully restraining patients also exposes the staff to risk of injuries. It has been reported from a large, state mental hospital in the USA, that one in five instances of mechanical restraint resulted in an injury to the patient or staff member involved.^35^

Since the foremost principle in clinical practice is not to harm our patients, these results advocate that use of mechanical restraint in modern psychiatry should be minimised. The observed absolute risk increases were low, but the derived relative risks were non-negligible when considering that less restrictive interventions are available. The results need to be considered at all levels of mental healthcare systems that still allow the use of mechanical restraint, as well as by decision makers in considering whether mechanical restraint should be fully or partly replaced by less restrictive alternatives.

## Supporting information

Baandrup and Kruse supplementary material Baandrup and Kruse supplementary material

## Data Availability

The data-set was made available for analysis on a national server and therefore cannot be shared.
