# Supplementary material for: Investigating the association of mechanical restraint with somatic harmful outcomes: national register-based study
Source: BJPsych Open. 2024 Nov 11;10(6):e205. doi: 10.1192/bjo.2024.799 (PMC11698205; doi:10.1192/bjo.2024.799)
Supplement: Baandrup and Kruse supplementary material [file S2056472424007993sup001.docx]

**Supplementary material**

**Detailed description of the statistical model**

Data was structured as an unbalanced panel, covering the period January 1^st^, 2007, to January 31^st^, 2019 (analysis of mortality until December 31^st^, 2019). In this panel, the following regression model was conducted:

$Y_{t}^{i} = {\alpha{}^{i}} + \beta FIX_{t}^{i}+ \gamma GROUP^{i} + \zeta X_{t}^{i} +\varepsilon$ (1)

Where Y is the outcome of interest, FIX is a dummy variable which is 1 only in the period from mechanical restraint and 30 days onwards (zero otherwise), GROUP is a time-invariant variable which is 1 for individuals that at some stage are subjected to mechanical restraint, and X is a vector of covariates comprising the following variables:

- Male= sex according to the population register
- Age= age in years measured at the time of restraint
- Treatment for alcohol abuse= at least one registration in the alcohol treatment register at any time
- Drug abuse= at least one registration in the drug abuse treatment register at any time
- Psychiatric diagnosis= a set of dummy variables for admissions with each section in ICD10 chapter F (*inter alia*, F0*, F1* etc.). Hence, an individual can have several psychiatric diagnoses. Only primary and secondary diagnoses are included
- Chronic, somatic illness= a set of dummy variables for having been diagnosed with asthma, COPD, dementia, diabetes type 1, diabetes type 2, rheumatoid arthritis, or osteoporosis
- Year of restraint/somatic outcome - included because there could be time trends affecting the incidence
- Municipality of residence - fixed effect term.

Regressions were conducted using xtreg in STATA, with clustered (robust) error terms, clustered at the individual level.

Model (1) was performed for each of the defined outcomes of interest: venous thrombosis and embolism (ICD-10 I26 pulmonary embolism, I74 arterial embolism and thrombosis, I80 phlebitis and thrombophlebitis, I82 other venous embolism and thrombosis), pneumonia (ICD-10 J18 pneumonia unspecified, J15 bacterial pneumonia, and J69.0 aspiration pneumonia), cardiac arrest (ICD-10 I46), mechanical injury (ICD-10 S00-99), and death from all causes. The coefficient of mechanical restraint $\beta$ can be interpreted as the within-individual difference whereas the group variable $\gamma$ can be interpreted as the between-group difference. Between-group results were defined as secondary outcomes.

Y was defined as the occurrence of a somatic harmful event within 30 days after the mechanical restraint. If an individual had more than one occurrence of restraint within 30 days, the exposure time was calculated as the time from the first occurrence to 30 days after the last occurrence.

The panel model specification ensured that we were able to observe the within-individual relationship of mechanical restraint with the somatic outcomes meaning that time-invariant possible confounding variables (e.g., sex, time since illness onset, pattern of behavior and of response to coercion) were controlled for within the design of the study. The full set of analyses were adjusted for age at event, sex, psychiatric diagnoses, chronic somatic illness, substance abuse, year of event, and municipality of residence.

In the dose-response analysis, only the mechanically restrained were included and dose-response was assessed in a linear regression:

$Y_{t}^{i} = {\alpha{}^{i}}_{d} +\beta_{d}Duration{}_{t}^{i} + \zeta_{d}X{}_{t}^{i} +\varepsilon$ (2)

where the outcomes are similar to equation 1, and subscript d denotes dose-response. Duration is a measure of the duration of the actual restraint, in days.

**Table S1**

Distribution of different types of coercion among the study population divided into exposed group (mechanical restraint) and control group (no mechanical restraint).

|  | Control group | Mechanical restraint group |
| --- | --- | --- |
| No compulsory admission | 11% | 17% |
| Compulsory admission | 89% | 83% |
| No involuntary treatment | 83% | 71% |
| Involuntary treatment | 17% | 29% |
| No physical restraint | 63% | 0% |
| Physical restraint excluding mechanical restraint | 37% | 51% |

**Table S2**

For each outcome the crude panel regression analysis is shown first followed by the full model adjusted panel regression analysis. All analyses expect mortality cover the period January 1^st^, 2007 to January 31^st^, 2019.

|  |  | Robust |  |  |  |  |
| --- | --- | --- | --- | --- | --- | --- |
| thrombo | Coefficient | std. err. | z | P>\|z\| | [95% conf. interval] | |
| coer_fix | .000161 | .0000783 | 2.06 | 0.040 | 7.66e-06 | .0003144 |
| group | -.0000127 | .0000237 | -0.54 | 0.592 | -.0000593 | .0000338 |
| _cons | .0000477 | .000017 | 2.81 | 0.005 | .0000144 | .000081 |

| thrombo |  | Robust |  |  |  |  |
| --- | --- | --- | --- | --- | --- | --- |
|  | Coefficient | std. err. | z | P>\|z\| | [95% conf. interval] | |
| mech_restr | .0001584 | .0000769 | 2.06 | 0.040 | 7.61e-06 | .0003092 |
| group | -9.80e-07 | .000025 | -0.04 | 0.969 | -.0000501 | .0000481 |
| male | 5.65e-06 | .0000237 | 0.24 | 0.812 | -.0000408 | .0000521 |
| age | 5.41e-07 | 5.19e-07 | 1.04 | 0.297 | -4.75e-07 | 1.56e-06 |
| Treatment for AUD | -.0000171 | .0000134 | -1.27 | 0.203 | -.0000435 | 9.21e-06 |
| Treatment for SUD  ≥1 hospital contact with a  diagnosis (ICD-10) of: | -.0000158 | .0000168 | -0.94 | 0.347 | -.0000486 | .0000171 |
| F00-F09 | .0000325 | .0000928 | 0.35 | 0.727 | -.0001494 | .0002143 |
| F10-F19 | .0000122 | .0000301 | 0.40 | 0.686 | -.0000469 | .0000712 |
| F20-F29 | -.0000429 | .0000609 | -0.70 | 0.482 | -.0001622 | .0000765 |
| F30-F39 | .0000677 | .0000434 | 1.56 | 0.119 | -.0000175 | .0001528 |
| F40-F49 | -5.57e-06 | .000033 | -0.17 | 0.866 | -.0000702 | .0000591 |
| F50-F59 | -.000021 | .0000128 | -1.64 | 0.102 | -.0000462 | 4.14e-06 |
| F60-F69 | -.0000248 | .0000117 | -2.12 | 0.034 | -.0000477 | -1.86e-06 |
| F70-F79 | -5.35e-06 | .0000219 | -0.24 | 0.807 | -.0000483 | .0000376 |
| F80-F89 | -1.32e-08 | .0000138 | -0.00 | 0.999 | -.000027 | .000027 |
| F90-F99  From the National Register of  Chronic Diseases: | -.0000133 | .0000204 | -0.65 | 0.517 | -.0000533 | .0000268 |
| Asthma | -.0000147 | .0000134 | -1.10 | 0.271 | -.000041 | .0000115 |
| Dementia | .0001804 | .0001836 | 0.98 | 0.326 | -.0001794 | .0005402 |
| COPD | -.0000148 | .0000164 | -0.90 | 0.366 | -.0000469 | .0000173 |
| Rheumatoid arthritis | -.0000178 | .000013 | -1.36 | 0.173 | -.0000433 | 7.81e-06 |
| Osteoporosis | -.0000402 | .0000213 | -1.89 | 0.059 | -.0000821 | 1.59e-06 |
| Diabetes_type1 | -.0000164 | .0000163 | -1.00 | 0.315 | -.0000483 | .0000156 |
| Diabetes_type2 | -7.28e-06 | .0000284 | -0.26 | 0.798 | -.0000629 | .0000484 |
| Year (somatic outcome) | 1.29e-06 | 3.03e-06 | 0.42 | 0.671 | -4.65e-06 | 7.23e-06 |

|  |  | Robust |  |  |  |  |
| --- | --- | --- | --- | --- | --- | --- |
| pneumonia | Coefficient | std. err. | z | P>\|z\| | [95% conf. interval] | |
| coer_fix | .0003111 | .0000853 | 3.65 | 0.000 | .0001439 | .0004783 |
| group | -.0000142 | .0000333 | -0.43 | 0.670 | -.0000795 | .0000511 |
| _cons | .0000928 | .0000199 | 4.66 | 0.000 | .0000537 | .0001319 |

|  |  | Robust |  |  |  |  |
| --- | --- | --- | --- | --- | --- | --- |
| pneumonia | Coefficient | std. err. | z | P>\|z\| | [95% conf. interval] | |
| mech_restr | .0002937 | .0000851 | 3.45 | 0.001 | .000127 | .0004604 |
| group | -.0000199 | .0000391 | -0.51 | 0.611 | -.0000965 | .0000567 |
| male | .0000479 | .0000401 | 1.19 | 0.232 | -.0000307 | .0001265 |
| age | 1.81e-06 | 1.44e-06 | 1.26 | 0.208 | -1.01e-06 | 4.62e-06 |
| Treatment for AUD | -.0000558 | .0000326 | -1.71 | 0.087 | -.0001198 | 8.16e-06 |
| Treatment for SUD  ≥1 hospital contact with a  diagnosis (ICD-10) of: | -.0000556 | .0000359 | -1.55 | 0.122 | -.000126 | .0000148 |
| F00-F09 | .0003543 | .000183 | 1.94 | 0.053 | -4.40e-06 | .000713 |
| F10-F19 | .000192 | .0001384 | 1.39 | 0.165 | -.0000792 | .0004633 |
| F20-F29 | -.0000585 | .0000985 | -0.59 | 0.552 | -.0002515 | .0001345 |
| F30-F39 | .0001452 | .0001229 | 1.18 | 0.237 | -.0000957 | .0003861 |
| F40-F49 | -.0001179 | .0000605 | -1.95 | 0.051 | -.0002365 | 5.99e-07 |
| F50-F59 | -.0000311 | .0000296 | -1.05 | 0.293 | -.0000892 | .0000269 |
| F60-F69 | -.0000528 | .0000242 | -2.18 | 0.030 | -.0001003 | -5.24e-06 |
| F70-F79 | -.0000692 | .000034 | -2.03 | 0.042 | -.0001359 | -2.53e-06 |
| F80-F89 | .0000539 | .0000565 | 0.95 | 0.341 | -.000057 | .0001647 |
| F90-F99  From the National Register of  Chronic Diseases: | -.0000874 | .0000524 | -1.67 | 0.095 | -.0001901 | .0000153 |
| Asthma | -.0000143 | .0000163 | -0.87 | 0.382 | -.0000462 | .0000177 |
| Dementia | -.0000879 | .0001816 | -0.48 | 0.628 | -.0004439 | .0002681 |
| COPD | .0002703 | .0003491 | 0.77 | 0.439 | -.000414 | .0009546 |
| Rheumatoid arthritis | -.000061 | .0000397 | -1.54 | 0.124 | -.0001388 | .0000167 |
| Osteoporosis | -.0001208 | .0000859 | -1.41 | 0.160 | -.0002891 | .0000475 |
| Diabetes_type1 | -.0000401 | .0000298 | -1.35 | 0.178 | -.0000986 | .0000183 |
| Diabetes_type2 | -.0000281 | .0000373 | -0.75 | 0.451 | -.0001011 | .000045 |
| Year (somatic outcome) | 1.48e-06 | 3.19e-06 | 0.46 | 0.643 | -4.78e-06 | 7.73e-06 |

|  |  | Robust |  |  |  |  |
| --- | --- | --- | --- | --- | --- | --- |
| cardiaca | Coefficient | std. err. | z | P>\|z\| | [95% conf. interval] | |
| coer_fix | .0000766 | .0000487 | 1.57 | 0.116 | -.0000189 | .000172 |
| group | -4.74e-06 | 4.35e-06 | -1.09 | 0.276 | -.0000133 | 3.78e-06 |
| _cons | 6.30e-06 | 4.30e-06 | 1.47 | 0.143 | -2.12e-06 | .0000147 |

|  |  | Robust |  |  |  |  |
| --- | --- | --- | --- | --- | --- | --- |
| cardiac arrest | Coefficient | std. err. | z | P>\|z\| | [95% conf. interval] | |
| mech_restr | .0000758 | .0000485 | 1.56 | 0.118 | -.0000193 | .0001709 |
| group | -.0000102 | 7.31e-06 | -1.40 | 0.162 | -.0000245 | 4.12e-06 |
| male | .0000122 | 9.26e-06 | 1.32 | 0.187 | -5.95e-06 | .0000304 |
| age | -3.49e-07 | 2.92e-07 | -1.20 | 0.232 | -9.20e-07 | 2.23e-07 |
| Treatment for AUD | -2.03e-06 | 2.92e-06 | -0.69 | 0.488 | -7.76e-06 | 3.70e-06 |
| Treatment for SUD  ≥1 hospital contact with a  diagnosis (ICD-10) of: | .0000278 | .0000263 | 1.06 | 0.291 | -.0000238 | .0000794 |
| F00-F09 | 8.99e-06 | 5.80e-06 | 1.55 | 0.121 | -2.38e-06 | .0000204 |
| F10-F19 | -7.44e-06 | 4.37e-06 | -1.70 | 0.089 | -.000016 | 1.12e-06 |
| F20-F29 | 6.97e-06 | 6.21e-06 | 1.12 | 0.262 | -5.21e-06 | .0000192 |
| F30-F39 | 4.10e-06 | 6.39e-06 | 0.64 | 0.521 | -8.42e-06 | .0000166 |
| F40-F49 | -3.92e-06 | 3.14e-06 | -1.25 | 0.212 | -.0000101 | 2.24e-06 |
| F50-F59 | -1.49e-06 | 1.74e-06 | -0.86 | 0.392 | -4.91e-06 | 1.93e-06 |
| F60-F69 | -3.15e-06 | 2.09e-06 | -1.51 | 0.132 | -7.24e-06 | 9.44e-07 |
| F70-F79 | -4.23e-06 | 2.08e-06 | -2.03 | 0.042 | -8.31e-06 | -1.49e-07 |
| F80-F89 | -2.66e-06 | 2.14e-06 | -1.24 | 0.214 | -6.86e-06 | 1.53e-06 |
| F90-F99  From the National Register of  Chronic Diseases: | 3.38e-06 | 4.68e-06 | 0.72 | 0.470 | -5.80e-06 | .0000126 |
| Asthma | 1.55e-06 | 3.43e-06 | 0.45 | 0.651 | -5.17e-06 | 8.28e-06 |
| Dementia | 4.53e-06 | .0000101 | 0.45 | 0.655 | -.0000153 | .0000244 |
| COPD | .0001562 | .0001278 | 1.22 | 0.222 | -.0000943 | .0004067 |
| Rheumatoid arthritis | -7.76e-06 | .0000115 | -0.67 | 0.500 | -.0000303 | .0000148 |
| Osteoporosis | -.000013 | 7.98e-06 | -1.63 | 0.102 | -.0000287 | 2.59e-06 |
| Diabetes_type1 | -1.70e-06 | 1.84e-06 | -0.92 | 0.356 | -5.30e-06 | 1.91e-06 |
| Diabetes_type2 | -4.34e-06 | 4.15e-06 | -1.05 | 0.295 | -.0000125 | 3.79e-06 |
| Year (somatic outcome) | -9.86e-09 | 2.11e-07 | -0.05 | 0.963 | -4.23e-07 | 4.03e-07 |

|  |  | Robust |  |  |  |  |
| --- | --- | --- | --- | --- | --- | --- |
| injury | Coefficient | std. err. | z | P>\|z\| | [95% conf. interval] | |
| coer_fix | .0004606 | .0001303 | 3.54 | 0.000 | .0002053 | .000716 |
| group | -.0000822 | .0000578 | -1.42 | 0.155 | -.0001955 | .0000312 |
| _cons | .0002784 | .0000506 | 5.50 | 0.000 | .0001793 | .0003776 |

|  |  | Robust |  |  |  |  |
| --- | --- | --- | --- | --- | --- | --- |
| injury | Coefficient | std. err. | z | P>\|z\| | [95% conf. interval] | |
| mech_restr | .0004005 | .0001286 | 3.11 | 0.002 | .0001484 | .0006526 |
| group | -.000165 | .0000786 | -2.10 | 0.036 | -.0003191 | -.0000109 |
| male | .0000992 | .0000962 | 1.03 | 0.302 | -.0000892 | .0002877 |
| age | -5.17e-06 | 2.98e-06 | -1.73 | 0.083 | -.000011 | 6.80e-07 |
| Treatment for AUD | .0001234 | .0001455 | 0.85 | 0.396 | -.0001617 | .0004085 |
| Treatment for SUD  ≥1 hospital contact with a  diagnosis (ICD-10) of: | -.0000533 | .0001435 | -0.37 | 0.710 | -.0003346 | .000228 |
| F00-F09 | .0007527 | .0002337 | 3.22 | 0.001 | .0002947 | .0012107 |
| F10-F19 | -.0001061 | .0001863 | -0.57 | 0.569 | -.0004713 | .000259 |
| F20-F29 | -.0002708 | .0001449 | -1.87 | 0.062 | -.0005549 | .0000132 |
| F30-F39 | .000095 | .0002553 | 0.37 | 0.710 | -.0004055 | .0005954 |
| F40-F49 | -.0000544 | .000164 | -0.33 | 0.740 | -.0003759 | .0002671 |
| F50-F59 | -.0007515 | .0006095 | -1.23 | 0.218 | -.0019462 | .0004431 |
| F60-F69 | .0005424 | .0002595 | 2.09 | 0.037 | .0000338 | .001051 |
| F70-F79 | -.0001682 | .0001828 | -0.92 | 0.358 | -.0005265 | .0001901 |
| F80-F89 | .0005349 | .000698 | 0.77 | 0.443 | -.0008332 | .001903 |
| F90-F99  From the National Register of  Chronic Diseases: | .0004093 | .0004598 | 0.89 | 0.373 | -.0004919 | .0013104 |
| Asthma | -.0000833 | .0001277 | -0.65 | 0.514 | -.0003336 | .0001669 |
| Dementia | .0003783 | .000218 | 1.74 | 0.083 | -.000049 | .0008056 |
| COPD | -.00004 | .0001195 | -0.34 | 0.738 | -.0002742 | .0001941 |
| Rheumatoid arthritis | -.0000883 | .0001969 | -0.45 | 0.654 | -.0004742 | .0002976 |
| Osteoporosis | .0007206 | .0008527 | 0.85 | 0.398 | -.0009507 | .0023918 |
| Diabetes_type1 | -.0001438 | .0000665 | -2.16 | 0.031 | -.0002742 | -.0000134 |
| Diabetes_type2 | -.0001502 | .0000594 | -2.53 | 0.011 | -.0002666 | -.0000338 |
| Year (somatic outcome) | .0000108 | 8.65e-06 | 1.25 | 0.213 | -6.17e-06 | .0000277 |

|  |  | Robust |  |  |  |  |
| --- | --- | --- | --- | --- | --- | --- |
| death | Coefficient | std. err. | z | P>\|z\| | [95% conf. interval] | |
| coer_fix | .0002627 | .0000219 | 12.00 | 0.000 | .0002198 | .0003056 |
| group | .0000479 | .0001522 | 0.31 | 0.753 | -.0002504 | .0003463 |
| _cons | .0003509 | .0000778 | 4.51 | 0.000 | .0001983 | .0005034 |

Analysis of death covers the period January 1st, 2007 to December 31st, 2019

|  |  | Robust |  |  |  |  |
| --- | --- | --- | --- | --- | --- | --- |
| death | Coefficient | std. err. | z | P>\|z\| | [95% conf. interval] | |
| mech_restr | .0002477 | .0000217 | 11.39 | 0.000 | .000205 | .0002903 |
| group | -7.95e-07 | .0000135 | -0.06 | 0.953 | -.0000272 | .0000256 |
| male | .0000211 | .0000204 | 1.03 | 0.303 | -.000019 | .0000611 |
| age | 5.20e-06 | 1.44e-06 | 3.62 | 0.000 | 2.39e-06 | 8.02e-06 |
| Treatment for AUD | .0000136 | 5.67e-06 | 2.40 | 0.016 | 2.48e-06 | .0000247 |
| Treatment for SUD  ≥1 hospital contact with a  diagnosis (ICD-10) of: | -.0000264 | 4.36e-06 | -6.06 | 0.000 | -.000035 | -.0000179 |
| F00-F09 | .0005516 | .0000148 | 37.20 | 0.000 | .0005226 | .0005807 |
| F10-F19 | -5.83e-06 | 5.69e-06 | -1.02 | 0.306 | -.000017 | 5.33e-06 |
| F20-F29 | -.0000346 | 4.98e-06 | -6.95 | 0.000 | -.0000444 | -.0000249 |
| F30-F39 | -.000042 | 5.69e-06 | -7.38 | 0.000 | -.0000532 | -.0000309 |
| F40-F49 | -.0000263 | 5.30e-06 | -4.96 | 0.000 | -.0000367 | -.0000159 |
| F50-F59 | 5.68e-06 | .0000116 | 0.49 | 0.624 | -.0000171 | .0000284 |
| F60-F69 | -.0000193 | 5.99e-06 | -3.22 | 0.001 | -.0000311 | -7.56e-06 |
| F70-F79 | -.000096 | .0000129 | -7.43 | 0.000 | -.0001214 | -.0000707 |
| F80-F89 | -.0000242 | .0000101 | -2.39 | 0.017 | -.0000441 | -4.37e-06 |
| F90-F99  From the National Register of  Chronic Diseases: | -.0000169 | 6.45e-06 | -2.62 | 0.009 | -.0000296 | -4.27e-06 |
| Asthma | 1.94e-06 | 9.46e-06 | 0.21 | 0.837 | -.0000166 | .0000205 |
| Dementia | .0002892 | .0000151 | 19.17 | 0.000 | .0002597 | .0003188 |
| COPD | .0001615 | .0000126 | 12.79 | 0.000 | .0001368 | .0001863 |
| Rheumatoid arthritis | -.0000717 | .0000269 | -2.66 | 0.008 | -.0001244 | -.0000189 |
| Osteoporosis | .0000999 | .0000132 | 7.56 | 0.000 | .000074 | .0001258 |
| Diabetes_type1 | .0002666 | .0000629 | 4.24 | 0.000 | .0001433 | .0003899 |
| Diabetes_type2 | .000031 | 7.34e-06 | 4.23 | 0.000 | .0000167 | .0000454 |
| Year (somatic outcome) | 7.44e-06 | 1.45e-06 | 5.12 | 0.000 | 4.59e-06 | .0000103 |

Analysis of death covers the period January 1^st^, 2007, to December 31^st^, 2019

|  |  |  |  |  |  |  |
| --- | --- | --- | --- | --- | --- | --- |
|  |  |  |  |  |  | |
|  |  |  |  |  |  |  |
|  |  |  |  |  |  |  |
|  |  |  |  |  |  |  |
|  |  |  |  |  |  |  |
|  |  |  |  |  |  |  |
|  |  |  |  |  |  |  |
|  |  |  |  |  |  |  |
|  |  |  |  |  |  |  |
|  |  |  |  |  |  |  |
|  |  |  |  |  |  |  |
|  |  |  |  |  |  |  |
|  |  |  |  |  |  |  |
|  |  |  |  |  |  |  |
|  |  |  |  |  |  |  |
|  |  |  |  |  |  |  |
|  |  |  |  |  |  |  |
|  |  |  |  |  |  |  |
|  |  |  |  |  |  |  |
|  |  |  |  |  |  |  |
|  |  |  |  |  |  |  |
|  |  |  |  |  |  |  |
|  |  |  |  |  |  |  |
|  |  |  |  |  |  |  |
|  |  |  |  |  |  |  |
|  |  |  |  |  |  |  |
|  |  |  |  |  |  |  |
